# Kinetics of CO_2_ Capture with Calcium Oxide during Direct Air Capture in a Fluidized Bed

**DOI:** 10.1021/acs.energyfuels.4c03770

**Published:** 2024-09-21

**Authors:** Bryan Kean Hong Ooi, Ewa J. Marek

**Affiliations:** Department of Chemical Engineering and Biotechnology, University of Cambridge, Philippa Fawcett Drive, CambridgeCB3 0AS, U.K.

## Abstract

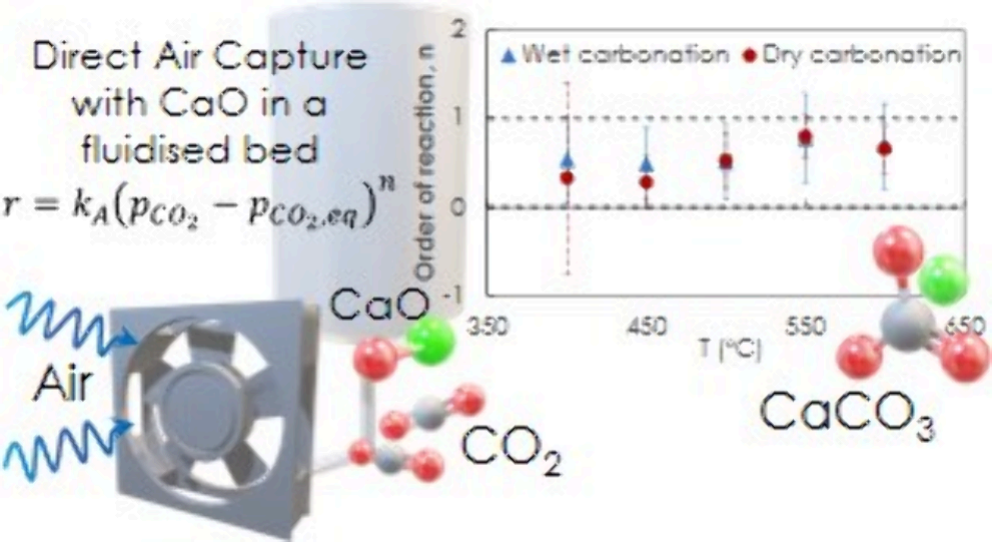

The bulk of research in carbon capture involves high CO_2_ concentrations. This work instead describes the kinetics of CaO carbonation in mixtures with *p*CO_2_ between 0.38 and 2.70 vol % and at temperatures between 400 and 650 °C. The reaction was studied in a bed of SiO_2_ fluidized at flow rates corresponding to *U/U*_mf_ of ∼4. Lower concentrations of CO_2_ connected to the rates of mass transfer of CO_2_ of the same order of magnitude as the rates of carbonation, thus, were eliminated from the kinetic analysis. The introduction of steam to the gas mixture (2 vol %) increased the rates of carbonation, demonstrating a pseudocatalytic effect, yet diminishing at higher temperatures. A rate expression generally accepted in the literature for high concentration CO_2_ in CaO carbonation was assessed for its applicability at near-equilibrium conditions, demonstrating that the order of the rate expression changes between 0 and 1, increasing at higher temperatures. Using nonlinear regression, the experimental values were fitted to the Langmuir–Hinshelwood rate expression. The obtained parameters indicate CO_2_ sorption and desorption being equilibrated, with the overall capture dominated mainly by slow kinetics of chemical reactions.

## 1 Introduction

The effects of anthropogenic CO_2_ on climate change are increasingly worrisome. With global temperatures setting record highs annually, the need for cost-effective solutions to actively remove CO_2_ from the atmosphere is ever more pressing.

Multiple solid sorbents have been proposed as potential candidates for CO_2_ capture, including CaO, MgO, alkali-metal oxides, and their modified versions designed to overcome certain material-related problems such as sintering.^1−4^ In general, these oxides react with CO_2_ to form carbonates in a reversible reaction. The alkali-based sorbents have been extensively studied for CO_2_ capture in high CO_2_ concentrations, simulating flue gas conditions from power plants, steel industry and cement production (up to 40,000 ppm of CO_2_). While necessary to design point-source carbon capture solutions, these studies omit lower CO_2_ concentrations, including the 2 orders of magnitude lower levels of CO_2_ in air (∼420 ppm of CO_2_).^5−8^ Addressing such a gap is needed to assess the potential of solid sorbents in Direct Air Capture (DAC), where CO_2_ is separated from ambient air.

The interest in CaO for DAC stems from its relatively high theoretical gravimetric uptake of 0.786 g_CO_2__ g_sorbent_^–1^ compared to other potential solid sorbents such as BaO with 0.288 g_CO_2__ g_sorbent_^–1^.^9^ However, the capacity of CO_2_ uptake with CaO is often lower than the stoichiometrically expected uptake, maintaining only ∼0.15 g_CO_2__ g_sorbent_^–1^ after hundreds of carbonation-decarbonisation cycles and the unavoidable material deactivation. The challenge of low CO_2_ uptake capacities will likely be encountered in realistic DAC applications^10^ but CaO remains a good candidate because of its application in the cement industry which can utilize the spent sorbent.

**Figure 1:**
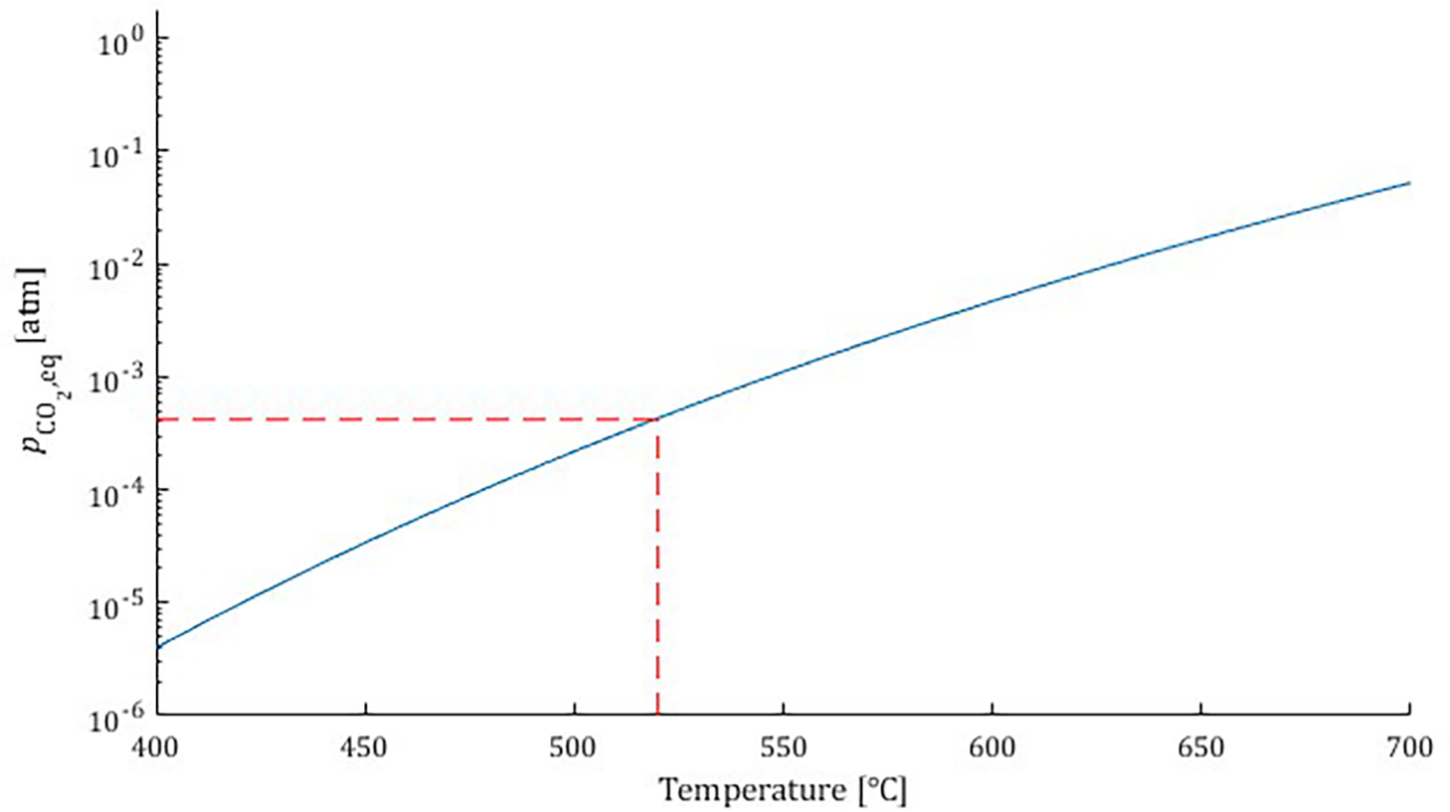
Equilibrium partial pressure of CO_2_, *p*_CO_2_,eq_ plotted against temperature. FactSage thermodynamics database was used to compute the *p*_CO_2_,eq_.^13^ Red dashed line indicates *p*_CO_2_,eq_ ≈ 420 × 10^–6^ atm, i.e. CO_2_ concentration in air.

Calcium oxide undergoes a reversible carbonation-calcination process, CaO+CO_2_ ⇌ CaCO_3_, also underpinning the technology called calcium looping. In the reversible loop, the forward or backward reactions dominate according to the thermodynamic constraints described by the equilibrium partial pressure of CO_2_, *p*_CO_2_,eq_, at a given temperature *T*. For CaO, the *p*_CO_2_,eq_ quickly approaches *p*_CO_2_, air_ which limits the CaO’s application for direct air capture to only low temperatures. The equilibrium *p*_CO_2_,eq_ as a function of temperature can be calculated taking from thermochemical data sets, e.g.:^11^
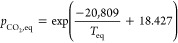
1where *p*_CO_2_,eq_ is in atm and *T*_eq_ is in K. The equilibrium curve of the CaO-CaCO_3_ system is illustrated in Figure 1. At 40,000 ppm of CO_2_ and atmospheric pressure, carbonation of CaO is thermodynamically favorable up to approximately 690 °C, compared to only 520 °C for 420 ppm of CO_2_ in air.

Most of the earlier works proposed that the surface reaction of CaO carbonation involves a two-step mechanism with the adsorption of CO_2_ onto the surface followed by the chemical reaction to form CaCO_3_,^1,12,13^ viz.
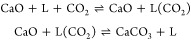
where L refers to an active site of CaO, and L(CO_2_) to the site occupied by CO_2_.

Experimentally, the rate of carbonation, *r* was then described by the power law as
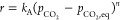
2where *k*_A_ is the rate constant and *n* is the order of reaction.

Sun et al. proposed that the CaCO_3_ formation and decomposition step is often equilibrated at low *p*CO_2_ values; thus, the adsorption step is rate-controlling.^12^ They found that *n* = 1 for cases with low driving force, i.e. when (*p*_CO_2__–*p*_CO_2_,eq_)<10 kPa, turning into *n* = 0 when the pressure difference exceeds ∼10 kPa, which the authors associated with the chemical reaction becoming rate-controlling.^12^ Multiple other authors confirmed *n* = 1 in eq 2; e.g. Yu and Fan gave *n* = 1 for (*p*_CO_2__–*p*_CO_2_,eq_) < 80 kPa at 700 °C,^13^ while Scaltsoyiannes et al. extended this result for (*p*_CO_2__–*p*_CO_2_,eq_) < 117 kPa between 670 and 820 °C.^1^ Similar studies are lacking for CaO reacting with CO_2_ at temperatures and concentrations relating to direct air capture. The broad range of the existing work suggests that for low driving force expected in DAC, i.e. *p*_CO_2__ ∼ *p*_CO_2_,eq_, we should expect *n* = 1.

Our work focuses on the CaO carbonation at low CO_2_ concentrations, similar to those in Direct Air Capture, to verify whether the expected rate expression remains a simple power-law with *n* = 1 at the unavoidably low driving forces. Additionally, we investigate the competing interplay between the driving force (concentration difference) for CO_2_ uptake, which is maximized for DAC with CaO at low temperatures, and the kinetic constant, *k*, which is low at low temperatures. Because both the driving force and the kinetic constant are strongly dependent on temperature, we performed the experimental work in a fluidized bed to ensure quick heat distribution. Similarly, because of the limited driving force for reaction, we assessed the potential limitations from CO_2_ delivery to ensure that the obtained rates of reactions remain in the kinetically controlled regime. This comprehensive approach adds to the discussion on CaO potential in direct air capture.

## 2 Experimental Section

Silica sand was used as a bed material. A batch of Fraction D sand obtained from David Ball Specialist Sands was washed and sieved to 200–250 μm. Similar size particles, 150–200 μm, of quicklime were obtained by grinding and sieving the Calbux commercial product from Lafarge Tarmac. The XRD analysis provided information about the composition of in quicklime particles: 96 wt % CaO, 4 wt % CaCO_3_ and negligible amounts of Ca(OH)_2_. The X-ray diffraction (XRD) profile for the quicklime used in this work is shown in Figure S1 in the Supporting Information (SI).

**Figure 2:**
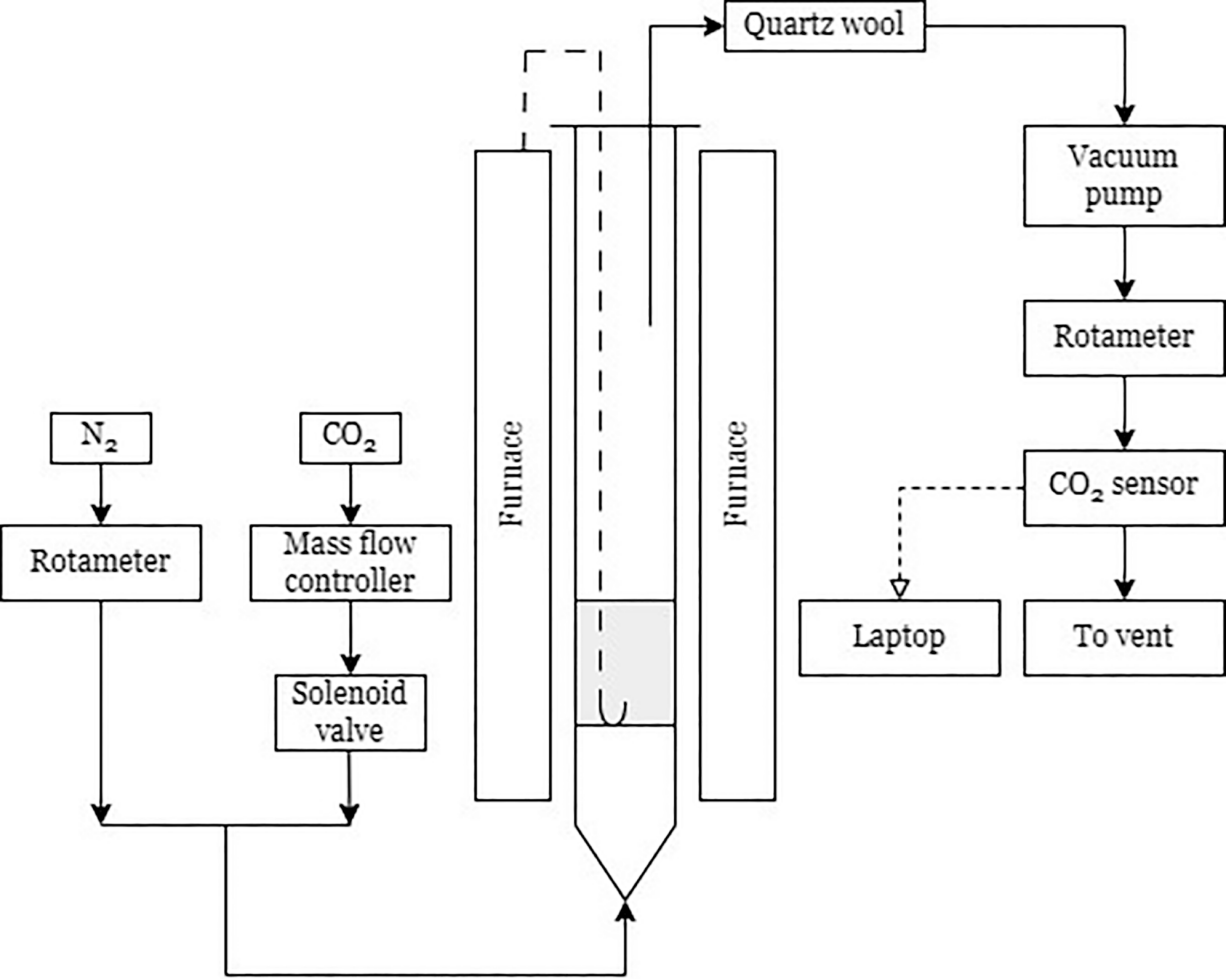
Schematics of the fluidized bed rig. Not illustrated are the desiccant and bubbler for wet carbonation. The desiccant was placed between the vacuum pump and quartz wool, while the bubbler was placed downstream of the mixing of N_2_ and CO_2_, and upstream of the inlet to the fluidized bed.

A schematic of the experimental setup is illustrated in Figure 2. Gases, N_2_ and CO_2_, were taken from the laboratory supply of reticulated gases with flow rates controlled with a rotameter and mass flow controller (Alicat MC-200SCCM), respectively. The flows were then connected and the resulting CO_2_–N_2_ mixture fed into an electrically heated quartz reactor (i.d. Thirty mm). The achieved concentrations of CO_2_ spanned from 0.38 to 2.70 vol %. The quartz tube contained a porous alumina disk, positioned ∼17 cm from the gas inlet. The disk held the solid particles of SiO_2_, used as the main bed material. A type K thermocouple measured the temperature ∼1 cm above the distributor, with readings used to control the temperature set point in experiments. The top of the reactor was covered with a loosely fitted plate to minimize air ingress. A sampling line was inserted from the top, with the sampling flow induced by a vacuum pump. The sample gas passed through a particulate filter, then a CO_2_ sensor (Gas Sensing Solutions SprintIR WHF-5) before being vented to the local exhaust.

Carbonation of CaO in the presence of steam was performed by bubbling the CO_2_–N_2_ gas mixture through a bubbler filled with water at 20 °C upstream of the inlet fluidized bed reactor. Calcium chloride (CaCl_2_) desiccant was then used to remove steam from the sampled gas stream before directing it to the CO_2_ sensor.

Experiments with 30 g of silica sand (200–250 μm) as the bed material were carried out at temperatures between 400 and 650 °C, with 50 °C increments. The gas flow rates were adjusted with temperature, keeping the ratio of *U/U*_mf_ ∼ 4. Samples of quicklime (∼20 mg, 150–200 μm) were introduced into the reactor from the top.

**Figure 3:**
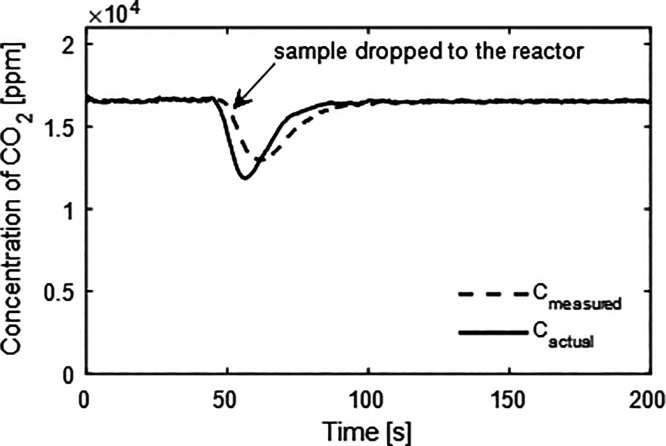
Example plot of CO_2_ concentration over time after introducing a batch of CaO sample. The lines present the measured concentration (*C*_measured_) and the deconvoluted result when accounting for mixing times in the sampling line (*C*_actual_).

The reaction between CO_2_ in the incoming gas and CaO was monitored by recording the CO_2_ depletion in the sampled gas. Each experiment was carried out three times. Exemplary results are presented in Figure 3. Initially, a constant concentration over time was observed, decreasing upon the introduction of the sample, when the CaO reacted with CO_2_. When the reaction finished, the CO_2_ concentration returned to the original value. The measured CO_2_ profiles were processed to account for mixing in the freeboard in the analyzer–the deconvolution procedure is described in the SI, Section 2.

During the experiments, the total molar flow rate through the reactor varied because of the CO_2_ uptake. In the experiments with steam, the flow rate that passed through the CO_2_ analyzer was dried, so the CO_2_ fraction was different to that in the reactor. To account for these differences, flow rates at the outlet were calculated by performing mass balances, as described in the SI, Section 2. The obtained consumption of CO_2_ over time, *F*_CO_2_,2_(*t*) in mol/s was normalized by the mass of CaO introduced, *m*_CaO_, arriving at the rate expression as . For assessing the kinetic parameters of carbonation, the maximum rates of reaction, *r*_max_ were taken, assuming the conversion of the CaO particle was, at that stage, negligible.

Thus, our analysis does not provide information on the rate dependency vs the CaO conversion. Such dependency, *f*(*X*), would describe how a particle of CaO reacts in the presence of the product CaCO_3_ and upon the whole duration of the process. Instead, here, we only focus on the starting moments for CaO carbonation. To check that all our data points indeed correspond to low *X*, we plotted the collected rates vs conversion in Figure S6 in the SI, confirming that all samples were analyzed at very low conversions, so our assumption of *X* ∼ 0 is justified.

To present how the conversion of CaO particles changes upon experiments, we plotted *X* vs time in Figure S7 of the SI. Because the collected signal for CO_2_ uptake was low, most of the observed CaO conversion corresponded to relatively large experimental errors. Taking *r*_max_ for assessing kinetics helps minimize the impact of these errors.

The conversion of CO_2_ introduced to the reactor was <20%, thus, for simplicity we assume a differential reactor. For a given temperature, a minimum of three sets of experiments were performed, varying the driving force (*p*_CO2_–*p*_CO2,e_). Each experiment was carried out three times.

## 3 Theory

The overall uptake and release of CO_2_ on calcium oxide can be written as
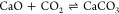
3

Most studies of CaO carbonation agree that the process employs a Langmuir–Hinshelwood mechanism^14,15^ involving a surface adsorption–desorption step (R1) and a chemical reaction step (R2) viz.
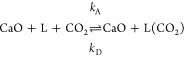
R1
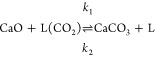
R2where L refers to unoccupied active sites, and L(CO_2_) refers to active sites occupied by adsorbed CO_2_.

Rates of the elementary steps in R1 and R2 are
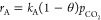
4
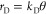
5
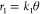
6
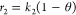
7where θ is the fraction of active sites occupied by CO_2_, and *k*_A_, *k*_D_, *k*_1_ and *k*_2_ refer to the rate constants of the adsorption, desorption, carbonation, and calcination steps, respectively.

In some works, R2 is replaced by a version presented as R3,^12^ where the creation of CaCO_3_ consumes the active site on the CaO surface, so the pool of L varies with particle conversion:
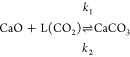
R3

Then, consequently, *r*_2_ = *k*_2_. Both mechanisms are often collapsed into a simplified rate expression, *r* = *k*_A_(*p*_CO_2__–*p*_CO_2_,eq_)^*n*^, where *n*, the order of reaction, varies from 0 to 1 and depends on the rate-limiting steps.

Sun et al. proposed that at low *p*_CO_2__, the chemical reaction step was rapid compared to the surface adsorption–desorption step.^15^ This meant that adsorbed CO_2_ molecules were rapidly consumed in the carbonation reaction to produce CaCO_3_, i.e. θ ≪ 1. Therefore, the surface adsorption–desorption step was rate controlling, i.e. *r* = *r*_A_ – *r*_D_
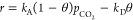
8

Substituting for θ and rearranging eq 8 yielded
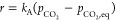
9

For the case where (*p*_CO_2__–*p*_CO_2_,eq_) had a zero-order relation with *r*, Sun et al. postulated that at a critical value of (*p*_CO_2__–*p*_CO_2_,eq_) ≈ 10 kPa, the fraction of active sites with adsorbed CO_2_, θ could no longer be assumed as low. In fact, they surmised that the driving force of CO_2_ for adsorption was high enough such that the active sites became mostly saturated, i.e. θ ≈ 1. At this stage, the chemical reaction step was rate-limiting. Thus, *r* = *r*_1_–*r*_2_ viz.
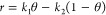
10
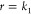
11

This means that the rate switches from the first order into a zero-order, depending on the driving force.

Interestingly, a different dominating mechanism was assumed by Ortiz et al.^15^ The authors state that gas–solid reactions are commonly limited by the chemical reaction step being slower than the adsorption step. Still, they arrive at the rate expression of the form similar to eq 9, where the driving force (*p*_CO_2__–*p*_CO_2_,eq_) had a first-order relation with *r* but the kinetic constant related to the chemical reaction step, *k*_1_.

For a small driving force (*p*_CO_2__–*p*_CO_2_,eq_), the process can quickly become limited by mass transfer rather than adsorption or chemical reactions. In a fluidized bed, the reacting solid CaO particles are distributed in the particulate, emulsion phase, while the gas with CO_2_ is divided between the bubble and particulate phases. An efficient exchange of gas between the two phases is needed, otherwise the reaction in the emulsion phase will lead to the local depletion of CO_2_, and the process might become limited by interphase mass transfer. The interchange of gas between bubbles and the particulate phase can be described by the crossflow factor, *X*, which describes the number of times the bubble volume is exchanged while a bubble moves through the bed. Here, the crossflow factor was assessed using the correlation developed by Davidson and Harrison^16^ using the potential flow theory.
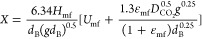
12where *d*_B_ is the diameter of a bubble, *H*_mf_ is the height of the bed at the minimum fluidization conditions, ε_mf_ is the bed voidage at minimum fluidization taken as 0.46. The solution for *X* requires finding the bed height, *H*, the mean diameter of bubbles, *d*_B_ and the velocity of bubbles, *U*_B_, simultaneously.^17^ The applied correlations are listed in the SI, Section 3.

For the described experiments, the crossflow factor was between 1.6 at 600 °C to 3.5 at 400 °C. The study by Hernández-Jiménez et al.^18^ assessed that the correlation for *X* based on the potential flow theory underestimates the values of *X*. Overall, the values of *X* here suggests a good mixing of gas between the two phases.

When the CaO particle is introduced to the emulsion phase, the CO_2_ for reaction needs to transfer through a boundary layer around the sorbent particle to its surface. Assuming a pseudosteady-state, the minimum rate of mass transfer through this boundary film can be calculated using Fick’s law for a dilute binary mixture of CO_2_ and N_2_ (expressed as species A and B, respectively),
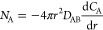
13where *r* represents the radial coordinate, *D*_AB_ is the binary diffusion coefficient between A and B, and  represents the concentration gradient of A in spherical coordinates. Eq 12 can be integrated with the boundary conditions at the particle surface (*r*_p_) and at the coordinate representing the distance from the particle surface to the end of the gas film (δ):
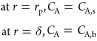
where *C*_A, s_ is the minimum concentration of CO_2_ on the surface of the particle taken as  at *T*, *C*_A, b_ is the bulk concentration of CO_2_ taken as  at *T*.
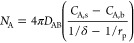
14

Normalizing *N*_A_ by the mass per particle of CaO,
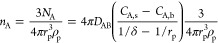
15where ρ_p_ is the mass density of CaO. The resulting units for *n*_A_ is mol s^–1^ g_CaO_^–1^. The thickness of the film layer was calculated after Hayhurst^24^ as , where ε_mf_ is the bed voidage at minimum fluidization taken as 0.46, and Sh is the Sherwood number, calculated as .

## 4 Results and Discussion

Before assessing the kinetic parameters, we screened the obtained results, eliminating the data points where the rate of CO_2_ delivery was less than an order of magnitude faster than the rate of carbonation, i.e. *n*_A_/*r*_max_ < 10. This eliminated a range of experiments at 500 and 600 °C when applying *p*_CO_2__ near ambient CO_2_ concentrations (∼420 ppm). This is because at higher temperatures the values of Re_mf_ are lower, leading to a lower flow rate of gas to reach the desired U/U_mf_. Moreover, for higher temperatures the rate of reaction, *r*_max_ increases exponentially with temperature. If DAC were to be performed in a bubbling fluidized bed above 400 °C, the process would likely be limited by external mass transfer.

**Figure 4:**
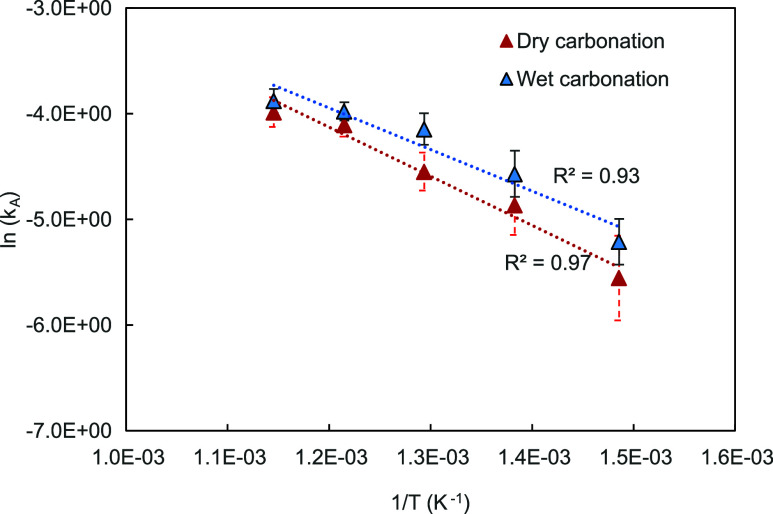
Plot of ln(*k*_A_) against 1/*T* where *k*_A_ was extracted by using eq 16, i.e. taking *n* = 1. The error bars indicate the 95% confidence intervals for each temperature data set (9 experiments).

Taking the rate expression as *r* = *k*_A_(*p*_CO_2__–*p*_CO_2_,eq_), i.e. eq 2 with *n* = 1, we can plot The values of *k*_*A*_ can be extracted by rearrangement
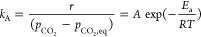
16where *E*_a_ is the activation energy, *A* is the pre-exponential factor, *R* is the ideal gas constant, and *T* is temperature. Then, the constants *A* and *E*_a_ can be computed from the Arrhenius plot, , presented in Figure 4.

From the intercepts and slopes, we obtained *E*_a_, which are higher than those reported previously in studies with high concentrations of CO_2_, when assuming *n* = 1, e.g. 22–29 kJ/mol in refs (1) and (12). On the other hand, Sun et al. predicted *E*_a_ of 41.5 kJ/mol, so close to the values in Figure 4, from the kinetic parameters of CaCO_3_ calcination reaction and the equilibrium constant. They ascribed the discrepancy to their experimentally obtained kinetics to the fact that equilibrium is likely not affecting the start of carbonation.^12^

**Figure 5:**
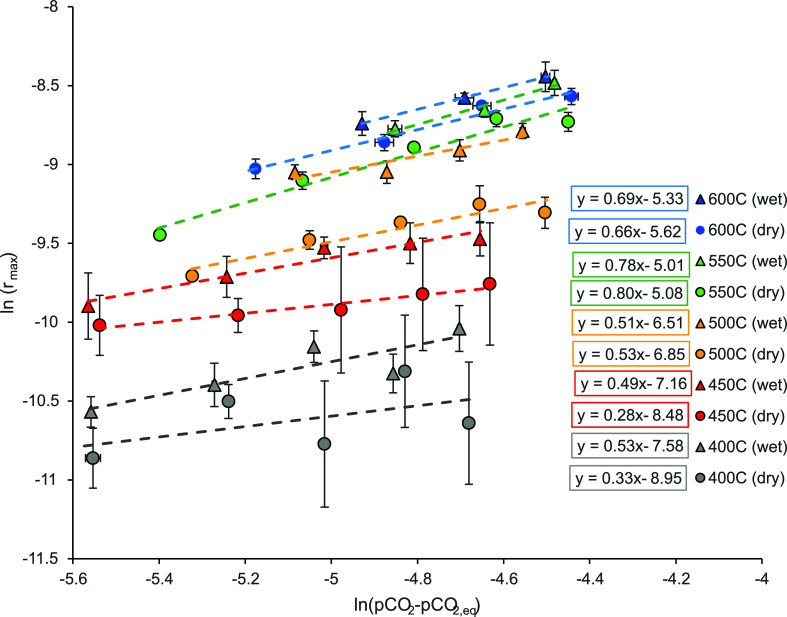
Plot of ln(*r*_max_) against ln(*p*_CO_2__–*p*_CO_2_,eq_) for all temperatures investigated after screening for externally mass transfer limited data points. The linear equations for each trend line are shown next to the corresponding temperature. The error bars depict the 95% confidence interval from three experimental points per (*p*_CO_2__–*p*_CO_2_,eq_).

From Figure 4, wet carbonation resulted in higher rates, and so *k*_*A*_ for across all temperatures investigated, with Figure 5 also demonstrating higher rates for all (*p*_CO_2__–*p*_CO_2_,eq_), as compared to dry carbonation, i.e. the pseudocatalytic effect. Manovic and Anthony reported that while steam enhanced the rate of carbonation, the effect was more pronounced at lower temperatures.^19^ However, this enhancement on the rate of carbonation was only observed in the diffusion-limited regime, i.e. the conversion of CaO to CaCO_3_ after *r*_max_. They attributed the enhanced rate of carbonation to the ability for steam to improve solid state diffusion through the CaCO_3_ product layer. The results in Figures 4 and 5 show the catalytic effects also in the kinetically controlled regime. However, the error bars depicting the 95% confidence intervals for dry and wet carbonation overlap for 400 and 450 °C. This indicates that the benefits of steam at 400 and 450 °C are not significantly pronounced. Moreover, the difference between *r*_max_ for wet and dry carbonation decreases with increasing temperature, further indicating diminishing returns from the addition of steam on *r*_max_ with increasing temperatures.

**Figure 6:**
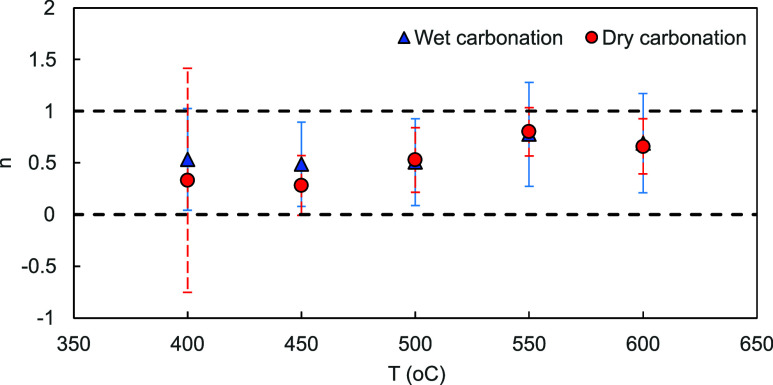
Plot of *n* against *T* for wet and dry carbonation for linear fitted lines in Figure 5. The black dashed lines illustrate both *n* = 1 and *n* = 0. The error bars indicate the 95% confidence intervals for each temperature data set.

Figure 5 gives a plot of ln(*r*_max_) against ln(*p*_CO_2__–*p*_CO_2_,eq_), and, recalling eq 2, provides the values for *n*–the order of reaction–directly from the slopes of linear fits. All values of *n* against the experimental temperatures are also presented in Figure 6. As discussed earlier, the governing rate expression for carbonation found in literature assumes that the order of reaction with respect to (*p*_CO_2__–*p*_CO_2_,eq_) is *n* = 1 for a broad range of (*p*_CO_2__–*p*_CO_2_,eq_), commonly defined as <10 kPa. However, the fitted linear equations and values for *n* in Figures 5 and 6 do not approach 1 across all temperatures. The range of (*p*_CO_2__–*p*_CO_2_,eq_) investigated here spans from 0.3 to 1.2 kPa–a significantly lower range than usually reported. To our knowledge, this is the first study to look into such low driving forces, assessing *n* and its behavior against *T*.

The behavior of *n* shifting between 0 and 1 here was unexpected, especially that the assumption of *n* = 1 led to a good linear fit and a small error range for *E*_A_ and *A* from the results in Figure 4. One potential reason for changes in *n* could be the nonvalidity of the assumptions made to derive the rate expression generally used in literature for carbonation but applied to much higher ranges of (*p*_CO_2__–*p*_CO_2_,eq_) than used in this work. Most work on carbonation assumes two-step processes–adsorption and chemical reaction. However, an earlier step may also be important. For example, Li et al.^20^ proposed that CaCO_3_ creation involves nucleation events, confirmed with atomic force microscopy. In their detailed model of the nuclei occurrence and growth, they considered a rate expression that describes the surface reaction, surface diffusion, as well as diffusion on grain boundaries and through the lattice, and, finally, the Ostwald ripening. Which of those steps could be limiting the rates in our experiments is beyond the scope of this work, but the fact that *n* changes quickly with *T* indicates that multiple phenomena might be at play simultaneously. We finally note that the presence of steam has little effect on *n*, further confirming that the chemical reaction might be limited by another intermediate step.

**Figure 7:**
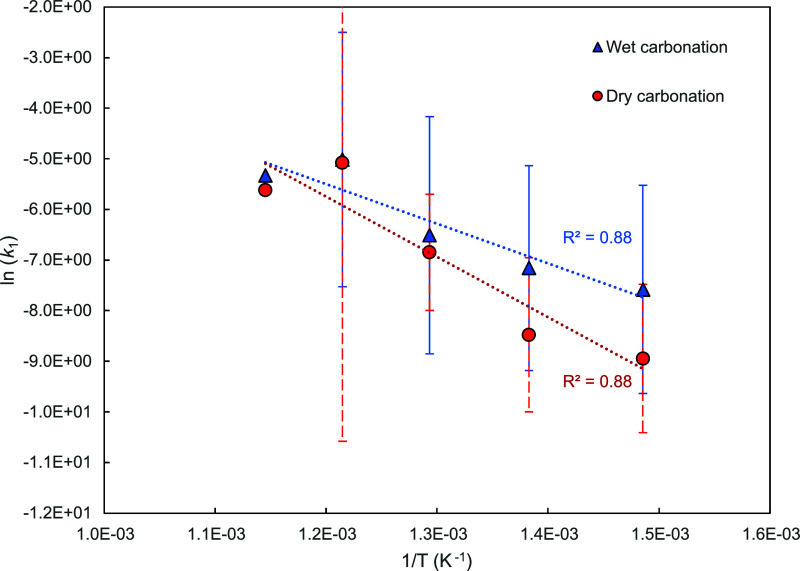
Plot of ln(*k*_A_) against 1/*T* for wet and dry carbonation, where ln(*k*_A_) was extracted from the *y*-axis intercepts of the linear equations in Figure 5. The error bars indicate the 95% confidence intervals for each temperature.

Taking the experimentally determined values for *n*, we recalculate *k*_A_ as  and use it again to assess the kinetic parameters. The results are presented in Figure 7. The achieved values for the activation energy are now significantly higher than in Figure 4, and closer to 77 kJ/mol obtained by Kyaw et al.^21^ Additionally, Ortiz et al.^15^ listed that the activation energy for the CaCO_3_ synthesis step is of the order of 20 kJ/mol, similar to the sorption steps, while the required activation energy for CaCO_3_ decomposition is closer to 180 kJ/mol. If the mechanism of carbonation, close to equilibrium, resulted from a mixture of the involved steps without specific limitation from any single one, then the *E*_A_ for the apparent *k*_A_ would range from 20 to 180 kJ/mol. Thus, overall, we conclude that the carbonation close to the equilibrium limitations, at low temperatures and driving forces represents a mixed multistep process.

Recalling from Section 3 that the overall calcium looping reaction CaO + CO_2_ ⇌ CaCO_3_ was proposed to follow the Langmuir–Hinshelwood mechanism with a surface adsorption–desorption step followed by a chemical reaction step, the rate expression can also be described using the full set of kinetic constants, eq 17.
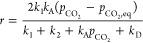
17
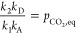
18

**Figure 8:**
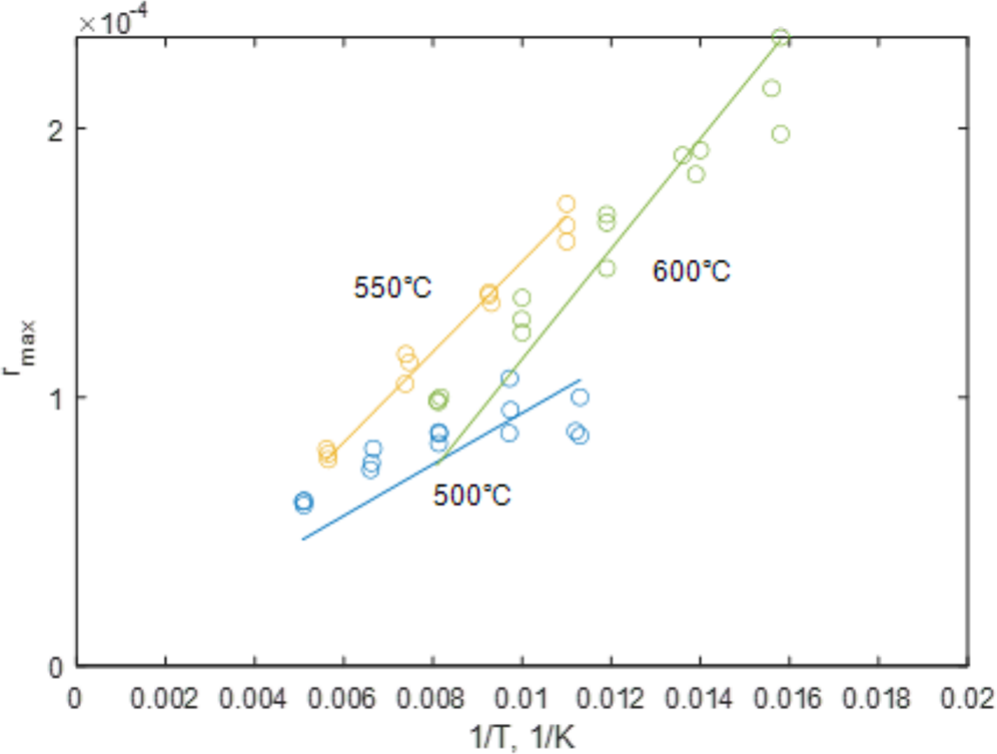
Plot of *r*_max_ against 1/*T*. The MATLAB *lsqnonlin* built-in function was used to fit *k*_A_, *k*_D_, *k*_1_, *k*_2_ to eqs 17 and 18 with the experimental *r*_max_ data.

From here, nonlinear fitting of *r* and *p*_CO_2__ can be performed to obtain *k*_1_, *k*_2_, *k*_A_ and *k*_D_, with the additional constraint from eq 18. The nonlinear fitting was performed using the *lsqnonlin* function in MATLAB while further constraining the solution values to be positive. Figure 8 shows the result of fitting the data points for *r*_max_ at various *p*_CO_2__ and temperatures for dry carbonation.

The parameters obtained as fits to the experimental results gave *k*_A_ ∼ *k*_D_. Only then, eq 18 was satisfied. We, thus, conclude that the adsorption–desorption step was at equilibrium, with the kinetics of the chemical reaction limiting the rate, as suggested by Moore for heterogeneous reactions.^22^ Assuming the Arrhenius form for *k*_1_ and *k*_2_, we obtained *E*_a,1_ = 4.5 kJ/mol for carbonation, while *E*_a,2_ = 31 kJ/mol for the reversed reaction. Both values are low, but their ratio, ∼ 7, is similar to that provided by Ortiz et al., i.e. Nine.^15^ The results thus suggest the dominating importance of the chemical reaction, possibly affected by additional phenomena, such as the appearance of nuclei of CaCO_3_.^20^

To finish the analysis, we finally estimate the degree in which the reaction was limited by intraparticle mass transfer, using the effectiveness factor, η.
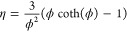
19where ϕ is the Thiele modulus:
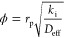
20

and *r*_p_ is the particle radius taken as the geometric mean, and *k*_i_ is the pseudo intrinsic first order rate constant in s^–1^. Next, the effective diffusivity *D*_eff_ is given as
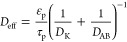
21where ε_p_ is the particle porosity taken as 0.5, and τ_p_ is the tortuosity taken as 2. Then, *D*_AB_ is the binary diffusion coefficient while the Knudsen diffusivity, *D*_K_ is given as
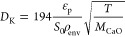
22where *S*_0_ is the specific surface area of the particle taken as 9.87 × 10^6^ m^2^ m^–3^ and ρ_env_ is the envelope density taken as 1.2 × 10^6^ g m^–3^,^23^ and *M*_CaO_ is the molar mass of CaO - 56 g mol^–1^.

Table 1 summarizes the estimated values of η for dry carbonation. Given that η ≈ 1 for all investigated conditions, intraparticle mass transfer was deemed a nonfactor, and thus, the values of *r*_max_ observed in this work were instead likely controlled by the surface reaction.

**Table 1:** Summary of η at the Temperatures Investigated

*T* [°C]	η	ϕ	*k*_i_ [s^–1^]
400	1.00	4.24 × 10^–07^	1.05 × 10^–10^
450	1.00	5.60 × 10^–07^	1.94 × 10^–10^
500	1.00	2.32 × 10^–07^	3.53 × 10^–11^
550	0.99	1.04 × 10^–07^	7.37 × 10^–12^
600	0.98	8.26 × 10^–08^	4.89 × 10^–12^

## 5 Conclusions

The carbonation of CaO in a fluidized bed was carried out to investigate the kinetics at near-equilibrium conditions. It was found that when the (*p*_CO_2__–*p*_CO_2_,eq_) driving force was between 1 × 10^–3^ atm to 2.5 × 10^–3^ atm, the carbonation reaction was limited by external mass transfer, thus higher CO_2_ concentrations were needed to study kinetics. The pseudocatalytic effect of steam accelerated carbonation in the kinetically controlled regime, albeit with diminishing returns with increasing temperature. At near-equilibrium conditions, the order of reaction, *n*, varied with temperature and did not reach *n* = 1, i.e. the value as commonly assumed in the literature. The obtained kinetic parameters, as well as the variations of *n* with *T* indicate the possible influence of phenomena connected to nucleation and the growth of nuclei. Nonlinear fitting of kinetic parameters to match the Langmuir–Hinshelwood expression demonstrated that the sorption step was equilibrated. In contrast, the kinetics of carbonation and decarbonation were rate-limiting for low-temperature CO_2_ capture, even at low investigated CO_2_ concentrations.

## Supporting Information Available

XRD results, details on data analysis, conversion of CaO particles, and fluidization parameters (PDF)
