# Supplementary material for: Kinetics of CO2 Capture with Calcium Oxide during Direct Air Capture in a Fluidized Bed
Source: Energy Fuels. 2024 Sep 21;38(22):22290–7. doi: 10.1021/acs.energyfuels.4c03770 (PMC11586902; doi:10.1021/acs.energyfuels.4c03770)
Supplement: Supplementary file 1 — ef4c03770_si_001.pdf [file ef4c03770_si_001.pdf]

Kinetics of CO_2 capture with calcium oxide during Direct Air Capture in a fluidised bed

Supplementary Information

Bryan Kean Hong Ooi[1], Ewa J. Marek[1*]

[1] Department of Chemical Engineering and Biotechnology, University of Cambridge, Philippa Fawcett Drive, CB3 0AS, United Kingdom

[*]Corresponding author(s): ejm94@cam.ac.uk

1. XRD results for the quicklime

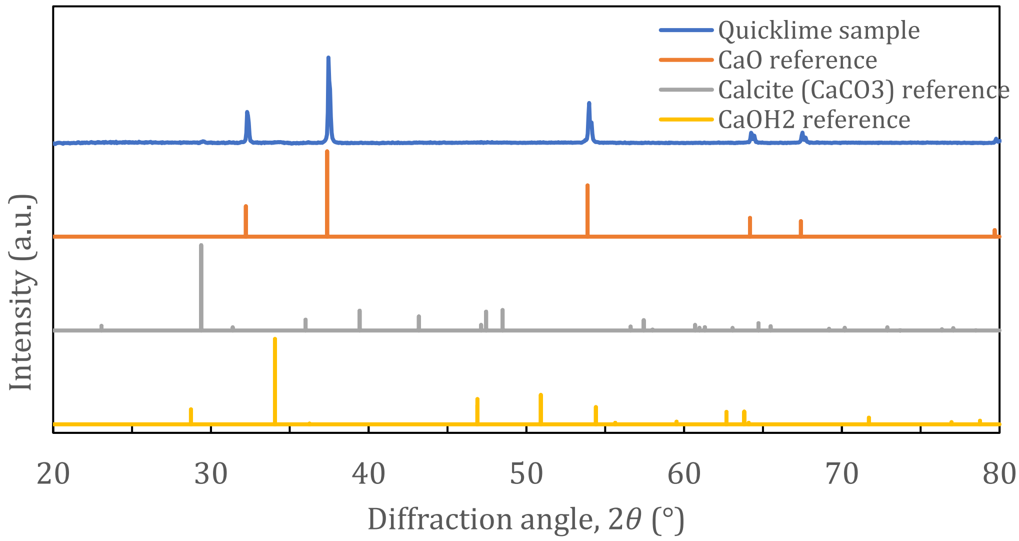

Fig. S1: XRD results for the quicklime sample and reference spectra for CaO, $CaCO_3$ (calcite) and $Ca(OH)_2$.

Figure 1 shows the XRD profile for the sieved quicklime used in this work along with the spectra for CaO, calcite ($CaCO_3$), and $Ca(OH)_2$. The refinement, performed with Profex provided information about the main components: ~96 wt.% CaO and ~4 wt.% $CaCO_3$.

2. Data analysis

<u>Molar balance for wet and dry carbonation</u>

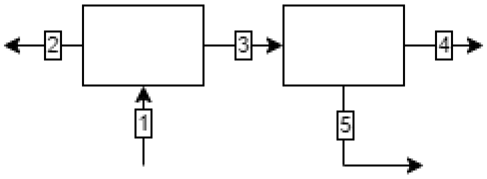

Fig. S2: Schematic diagram for molar balance. Stream (1) refers to the reactor inlet, stream (2) refers to the consumption of CO_2 during the carbonation reaction, stream (3) is the vent, stream (4) is the CO_2-N_2 portion of the vent, and stream (5) is the H_2O portion of the vent.

Figure S2 depicts a schematic diagram used for molar balance. Since N_2 was assumed to be inert, $F_{\mathrm{N_2},1} = F_{\mathrm{N_2},4} = F_{N_2}$. Two key variables, the inlet CO_2 concentration, $y_{CO_2,1}$ and the consumption of CO_2 during carbonation $F_{\mathrm{CO_2},2}(t)$ were to be extracted from the molar balance. The inlet CO_2 concentration was desired since it was assumed that the bulk concentration of CO_2 during reaction equalled the inlet CO_2 concentration.

Firstly, for wet carbonation, the molar fraction of steam at the reactor inlet, $y_{H_2O,1}$ was assumed to be saturated at 20℃ calculated by the Antoine equation *viz.*

$$\log_{10} p^*_{\mathrm{H_2O}}(T) = 5.40221 - \frac{1838.675}{-31.737 + T} \tag{1}$$

where $p^*_{\mathrm{H_2O}}(T)$ is the vapour pressure of water in bar at temperature T in K, and the constants were taken from the National Institute of Standards and Technology (NIST) database [31]. Thus, the vapor pressure of water in experiments with a bubbler at 20℃ was 0.023 atm. For dry carbonation, $y_{H_2O,1} = 0$. Next, the instantaneous molar fraction of CO_2 at the outlet measured by the CO_2 sensor, $y_{CO_2,4}(t)$ was expressed and rearranged as

$$y_{CO_2,4}(t) = \frac{F_{CO_2,4}(t)}{F_{CO_2,4}(t) + F_{N_2}} \tag{2}$$

$$F_{CO_2,4}(t) = \left(\frac{y_{CO_2,4}(t)}{1 - y_{CO_2,4}(t)}\right) F_{N_2} \tag{3}$$

The inlet molar fraction of steam, $y_{H_2O,1}$ was taken as

$$y_{H_2O,1} = \frac{F_{H_2O,1}}{F_{H_2O,1} + F_{CO_2,1} + F_{N_2}} \tag{4}$$

Rearranging in terms of $F_{H_2O,1}$ yielded

$$F_{H_2O,1} = \left(\frac{y_{H_2O,1}}{1 - y_{H_2O,1}}\right)\left(F_{N_2} + F_{CO_2,1}\right) \tag{5}$$

Subsequently, by overall balance of CO_2,

$$F_{CO_2,2}(t) = F_{CO_2,1} - F_{CO_2,4}(t) \quad (6)$$

Before CaO was introduced into the reactor, no reaction occurred, *i.e.* $F_{CO_2,2}(t) = 0$. Therefore, $F_{CO_2,1}$ was extracted by considering the setup before CaO was introduced into the system, *i.e.* $F_{CO_2,1} = F_{CO_2,4}(t)$. Finally, $y_{CO_2,1}$ was calculated as

$$y_{CO_2,1} = \frac{F_{CO_2,1}}{F_{H_2O,1} + F_{CO_2,1} + F_{N_2}} \quad (7)$$

For dry carbonation, $F_{H_2O,1} = 0$.

<u>Raw data extraction and processing</u>

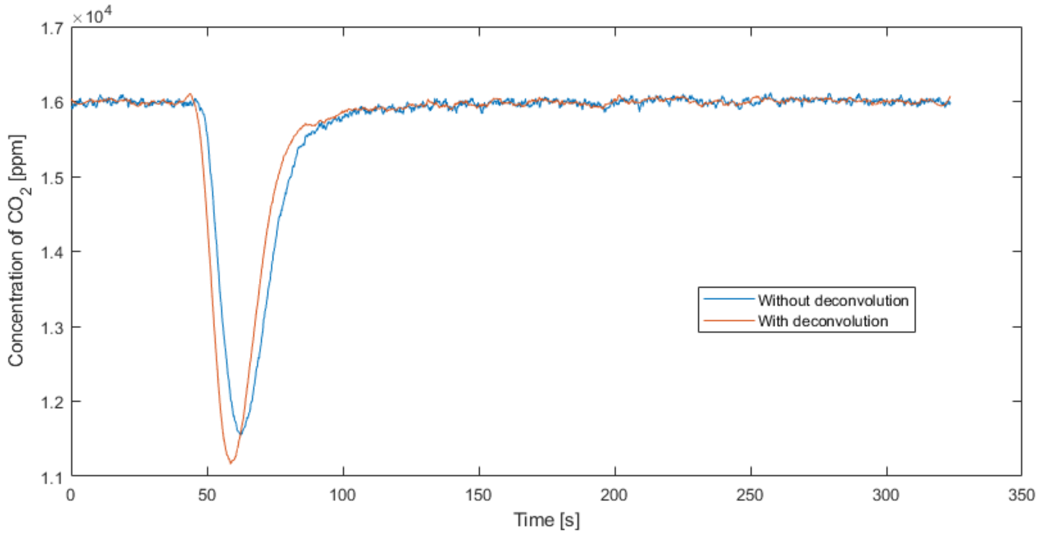

Fig. S3: Example plot of CO_2 concentration over time after introduction of CaO.

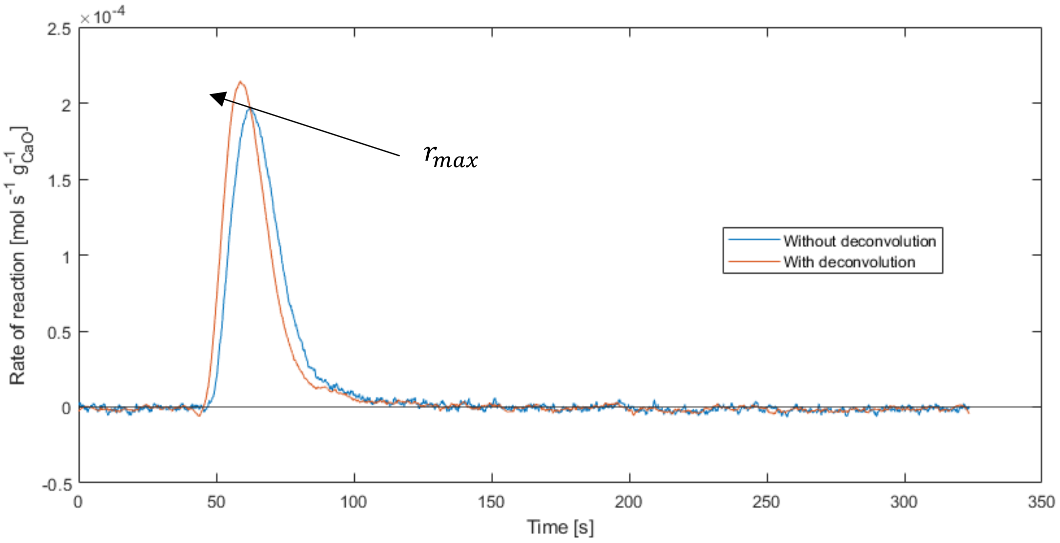

Fig. S4: Example plot of rate of reaction over time. Also illustrated is r_{max}.

Figure S3 illustrates a typical profile for CO_2 concentration over time observed in this study. Deconvolution was performed to computationally remove the effects of measurement by modelling the mixing in the fluidised bed and the freeboard, and the mixing in the analyser as two continuous stirred tank reactors (CSTRs) in series with mixing times, τ_{bed} and τ_0, respectively. To deconvolute the results obtained in the experiments, the measured concentration of CO_2, C_m was fitted into C_{fitted} in (3-8):

$$C_{\mathrm{fitted}} = C_0 + (C_1 - C_0)\left(1 - \exp\left[\frac{t}{\tau_m}\right]\right) \tag{8}$$

where C_0 is the concentration of CO_2 before a step change was introduced, C_1 is the concentration of CO_2 after the step change, t is the current time, and τ_m is the mixing time of the system. For two CSTRs in series (ignoring second order effects), $\tau_{bed} = \tau_m - \tau_0$. The best fit for τ_m was computed by minimising the least squared error between C_{fitted} and C_m in MATLAB, using the *lsqnonlin* built-in function illustrated in Fig. S5.

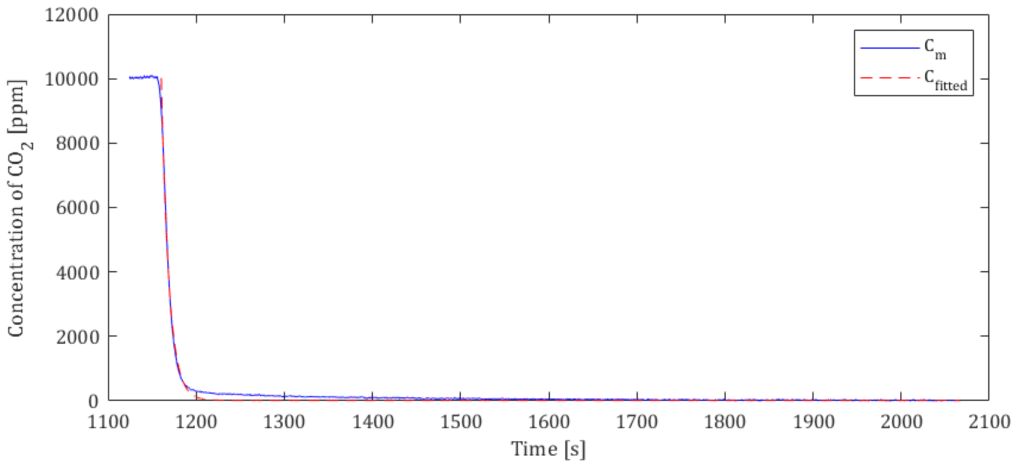

Fig. S5: Example plot of concentration against time for C_m and C_{fitted}.

After τ_{bed} was obtained, the deconvoluted concentration profile of CO_2 in the bed, C_{actual} was computed as:

$$C_{\mathrm{actual}}(t) = C_{\mathrm{m}}(t) + \tau_{bed}\frac{dC_{\mathrm{fitted}}}{dt} \tag{9}$$

3. Conversion *vs* time of experiments

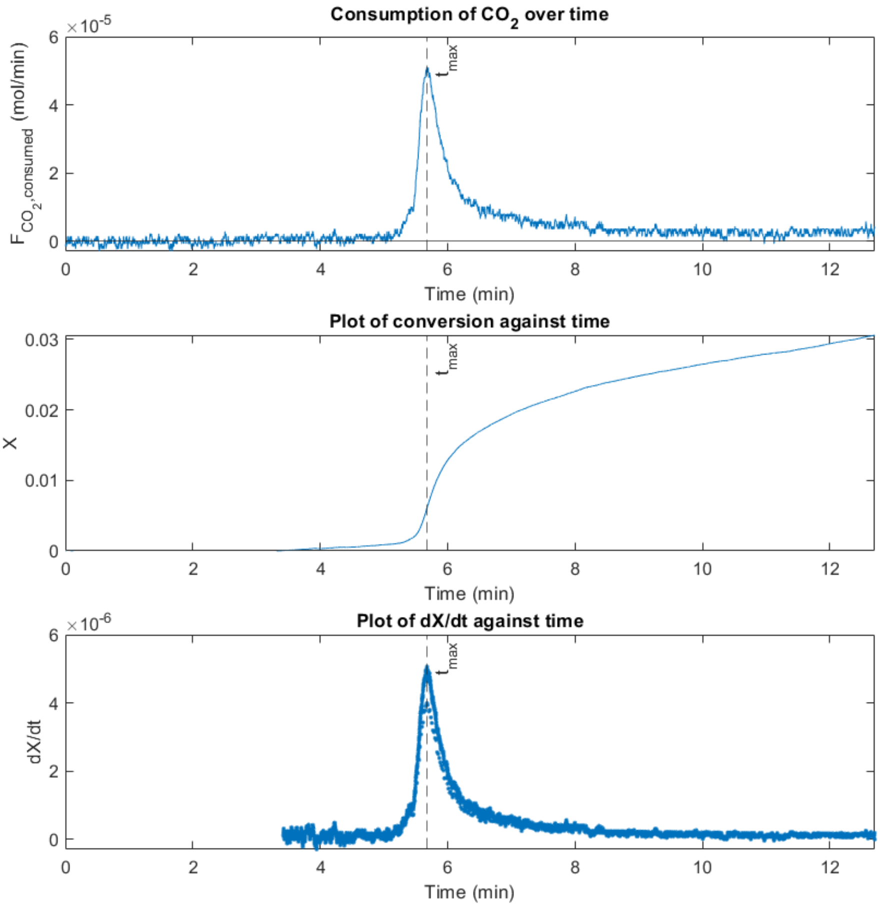

Fig. S6: Exemplary experiment for CO_2 uptake (a) rate of the CO_2 removal in time of experiment, (b) change in conversion *vs* time of experiment, (c) rate of carbonation of experiment. The final conversion of 0.1016g CaO (0.0018 mol CaO) was 3.2% over approximately 20 minutes.

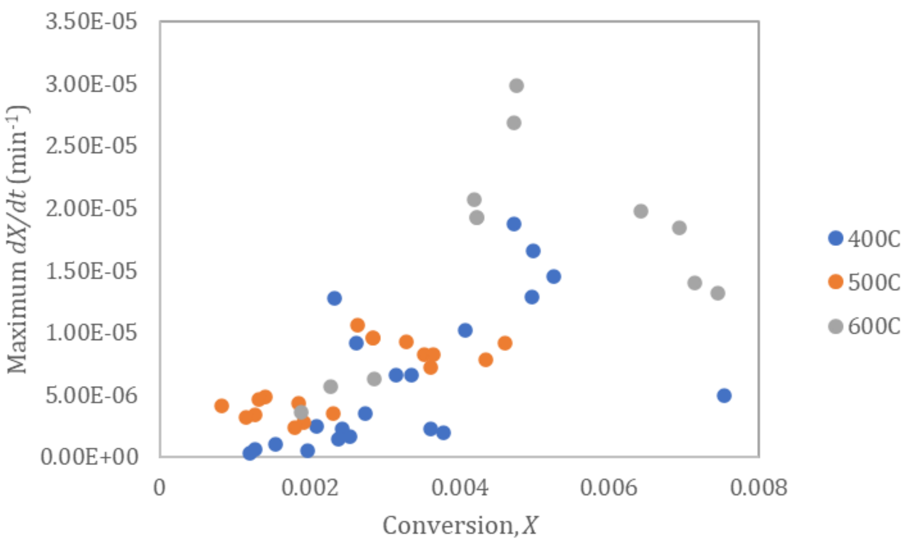

Fig. S7: Maximum rate (*dX/dt*) against CaO conversion (*X*). Variations across a given temperature were linked to changing the reaction driving force ($p_{CO2} - p_{CO2,eq}$), which was controlled in the experiments..

4. Fluidisation parameters

The average size of bubbles was calculated as:

$$d_B = 0.54\left(U - U_{mf}\right)^{0.4}\left(H + 4\sqrt{A_d}\right)^{0.8} g^{-0.2} \tag{10}$$

where Ad is the area of the distributor per orifice, here taken as 0 for a porous sintered plate. To calculate d_B we took $H = H_{mf}$ as the hight at which the average bubble size occurs. Then, we used the connection between U, H ε as follow:

$$\varepsilon_B = \frac{H - H_{mf}}{H} = \frac{U - U_{mf}}{U_B} \tag{11}$$

Where U_B is the velocity of bubbles, given by Davidson and Harrison:

$$U_B = 0.711\sqrt{g d_B} \tag{12}$$
